# Fully Customized Photoacoustic System Using Doubly Q-Switched Nd:YAG Laser and Multiple Axes Stages for Laboratory Applications

**DOI:** 10.3390/s22072621

**Published:** 2022-03-29

**Authors:** Unsang Jung, Jin Hyuck Choi, Han Tae Choo, Gyu Ug Kim, Jaemyung Ryu, Hojong Choi

**Affiliations:** 1 Production Technology Research Center, Kumoh National Institute of Technology, 61 Daehak-ro, Gumi 39177, Gyeongsangbuk-do, Korea; usjung@kumoh.ac.kr; 2 Department of Physics, Kumoh National Institute of Technology, 350-27 Gumi-daero, Gumi 39253, Gyeongsangbuk-do, Korea; jh-choi@kmoh.ac.kr; 3 Department of Optical Engineering, Kumoh National Institute of Technology, 350-27 Gumi-daero, Gumi 39253, Gyeongsangbuk-do, Korea; htchoo@kmoh.ac.kr (H.T.C.); gukim@kumoh.ac.kr (G.U.K.); 4 Department of Electronic Engineering, Gachon University, Seongnam-daero, Sujeong-gu, Seongnam 13420, Gyeonggi-do, Korea

**Keywords:** doubly Q-switch, photoacoustic system, customized laser system

## Abstract

We developed a customized doubly Q-switched laser that can control the pulse width to easily find weak acoustic signals for photoacoustic (PA) systems. As the laser was constructed using an acousto-optic Q-switcher, in contrast to the existing commercial laser system, it is easier to control the pulse repetition rate and pulse width. The laser has the following control ranges: 10 Hz–10 kHz for the pulse repetition rate, 40–150 ns for the pulse width, and 50–500 μJ for the pulse energy. Additionally, a custom-made modularized sample stage was used to develop a fully customized PA system. The modularized sample stage has a nine-axis control unit design for the PA system, allowing the sample target and transducer to be freely adjusted. This makes the system suitable for capturing weak PA signals. Images were acquired and processed for widely used sample targets (hair and insulating tape) with the developed fully customized PA system. The customized doubly Q-switched laser-based PA imaging system presented in this paper can be modified for diverse conditions, including the wavelength, frequency, pulse width, and sample target; therefore, we expect that the proposed technique will be helpful in conducting fundamental and applied research for PA imaging system applications.

## 1. Introduction

Ultrasound systems are widely used to obtain structural image data in deep areas of approximately 10 cm; thus, they have high spatial resolutions but low contrast resolutions owing to tissue acoustic properties [1,2,3]. An optical system is used to obtain physiological image data [4,5]. Due to the characteristics of light, the limitation depth of the optical system is smaller than that of the ultrasound system, but the contrast resolution of the optical system is significantly higher than that of the ultrasound system [6]. Ultrasound and optical systems transmit/receive ultrasound energy and light or power, respectively [7]. The photoacoustic (PA) system is a hybrid system of the optical and ultrasound systems; thus, it is constructed wave by the absorbed light and receives an ultrasound system, and it is possible to produce structural and physiological data [8]. The major light source in the PA system is light amplification due to stimulated emission of radiation (laser), which generates vibration energy from the target, and acoustic energy is detected by the ultrasound transducer to show the image data [9,10].

PA systems are divided into two major categories: PA tomography and microscopy systems [11,12]. In a PA tomography system, light is diffused and converted into transient thermal energy in a large target area [9]. The acoustic thermal waves are then detected using a non-focused ultrasound transducer. In a PA microscopy system, the light is focused through the optical lens and delivered to a small target area [13]. The reflected acoustic waves were received by a focused ultrasound transducer. Therefore, the PA microscopy system can provide a higher spatial resolution of the desired target than the PA tomography system. To cover a large area in the PA microscopy system, a mechanical motor is used to support the light source or ultrasound transducer [8]. The resolution of the motor affects the spatial resolution of the PA system [13].

The quality of a PA system depends on how well light is generated and the ultrasound signal is received [14]. To develop a PA system, proper light-generation techniques must be developed, because the laser component is one of the most crucial components to be constructed [8,15]. Light-emitting diodes (LEDs) are also used as light sources in PA systems. However, the LED is a divergent light source; thus, it must use low-aberration and complex optical system components [16]. Additionally, the LED light wavelength is shorter than that of the laser [17]. In a PA system, the acoustic waves reflected by the tissue target have 0.1% of the energy of those for the ultrasound-only system; thus, properly customized light generation is challenging [11,18]. Therefore, the laser is preferable to the LED as a light source in a PA system.

Laser operation using Nd:YAG (neodymium:yttrium aluminum garnet, Y_3_Al_5_O_12_) crystals was first demonstrated by J. E. Geusic at Bell Labs in 1964. Nd:YAG crystals have been used in lasers that have received considerable attention owing to their high gain, low threshold, high efficiency, low loss at 1064 nm, excellent thermal conductivity, thermal shock, and various oscillation wavelengths [19,20]. The main oscillation wavelengths of the Nd:YAG laser are 946, 1064, and 1319 nm, of which 1064 nm has the highest gain. Using a four-level Nd:YAG laser, lamp pumping or pumping using a diode laser is possible. In the laboratory, an 808 nm laser diode (LD) with a small size, high efficiency, and stable continuous pulse oscillation can be applied in various studies. In particular, laboratory-fabricated active and passive Q-switched nanosecond pulsed Nd:YAG lasers with low average power are used for micromachines, optical data storage, coherent light source, and communication applications and have recently been widely employed as light sources for PA image implementation using PA effects.

Until now, there has been no commercial product of the doubly Q-switched laser source that can change the pulse width. Compared with ultrasound-only systems, in the PA system, pulse width variance is helpful for finding weak acoustic signals. Therefore, we developed a customized doubly Q-switched laser source for PA systems. The wavelength of the laser used in the PA system is between 400 and 1600 nm [21]. In particular, visible light to near-infrared with wavelengths between 600 and 1200 nm is utilized, because of the relatively large penetration depth of living tissues [16,17]. Therefore, we propose a 1064 nm fully customized laser source for a PA system. In addition, a multi-modular multi-axis stage optimized for laboratory research was fabricated and a PA system was implemented using the developed laser. The usability of the PA system was verified through an imaging test of hair and insulating tape.

The proposed doubly Q-switched 1064 nm Nd:YAG laser has a cost similar to that of a commercial laser (USD 8000–USD 20,000), but is tailored to PA imaging. The advantage of this laser is that due to the characteristics of the Nd:YAG laser, it is possible to convert the oscillation wavelength through some modifications of the optical device without additionally purchasing a laser of a different wavelength. In addition, the developed laser can control the performance (pulse energy and output power, pulse repetition rate, pulse width) required for photoacoustic technology research, so it is more efficient than commercial lasers in research efficiency.

Section 2 describes the construction of a customized doubly Q-switched laser that can control the pulse width and support the sample stage system. In Section 3, PA images measured using a hair phantom are presented to confirm the feasibility of the developed laser system. Finally, Section 4 presents the conclusions.

## 2. Materials and Methods

### 2.1. Doubly Q-Switched 1064 nm Nd:YAG Laser Configuration

In this section, we present the detailed development of the customized laser system. 

**Figure 1:**
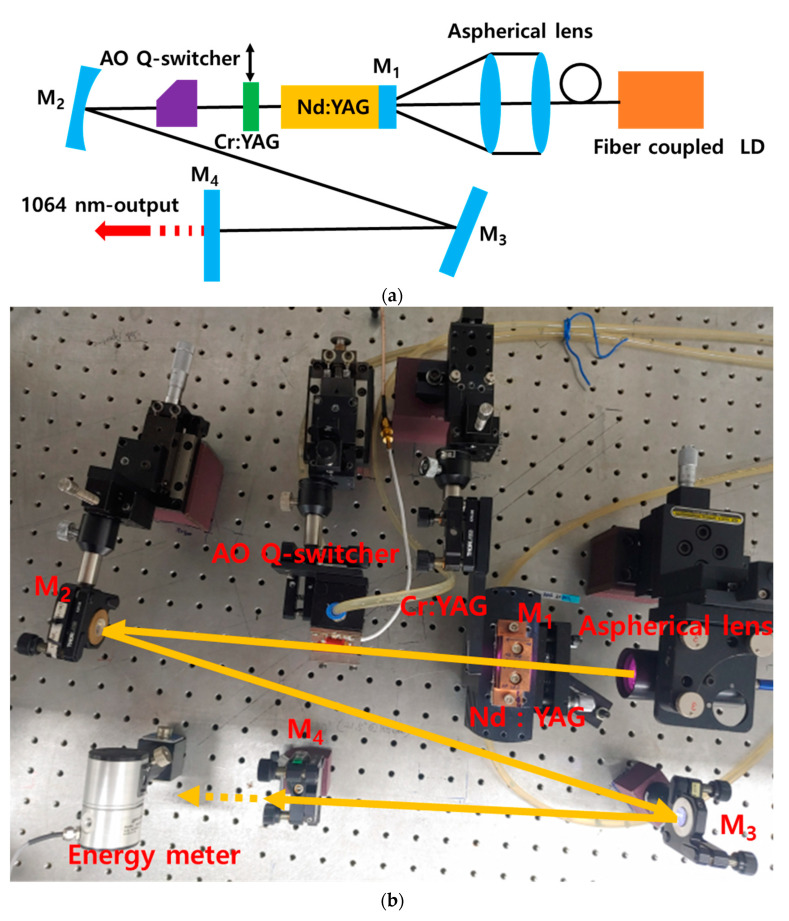
(**a**) Schematic of the developed doubly Q-switched Nd:YAG laser and (**b**) the implemented laser optical system.

Compared with the passive Q-switched nanosecond pulsed Nd:YAG laser using a Cr:YAG saturable absorber, an active Q-switched nanosecond pulsed Nd:YAG laser using an acousto-optic (AO) device is used as a light source for PA applications that can control the pulse repetition rate. Therefore, it is often used as a light source for PA imaging applications. In this study, a stable Q-switched nanosecond pulsed Nd:YAG laser with an adjustable repetition rate was developed for PA imaging at a lower cost than the existing laser (approximate price) used for PA imaging applications. The pulse width of a Q-switched Nd:YAG laser using an AO device is usually several hundred nanoseconds. In a recent study, a shorter pulsed light source was needed to realize PA imaging [8]. Therefore, the Q-switched Nd:YAG laser developed in this study is not capable of adjusting the pulse repetition rate, but a Cr:YAG saturable absorber with a shorter pulse width and an AO device capable of adjusting the pulse repetition rate was used to develop a doubly Q-switched Nd:YAG laser. Figure 1a,b shows the resonator structure and implemented system of the doubly Q-switched Nd:YAG laser developed for this study.

The pulse width of the AO Q-switched laser is generally long, and the temporal pulse output shape is asymmetric; thus, the pulse rise time is short, but the fall time is long. However, more symmetrical pulses are required for PA images. As mentioned previously, compared with AO Q-switched lasers, passive Q-switched lasers can produce shorter pulses, but the pulse repetition rate is not stable, and the pulse peak power is low. To address these issues, a short pulse with high peak power and stable repetition rate can be generated by inserting an AO device and a saturable absorber Cr:YAG into the resonator for double Q-switching [22,23,24,25,26,27]. To obtain a stable Nd:YAG laser with a wavelength of 1064 nm, single-sided pumping with an LD having a wavelength of 808 nm was performed using an AO Q-switch device and a Cr:YAG saturated absorber. Therefore, we developed a Nd:YAG laser with a doubly Q-switched Z-type resonator structure. As the pumping laser, an LD (DS3-41322-111, BWT Inc., Beijing, China) capable of generating light with a wavelength of 808 nm and a maximum output of 100 W was used. An optical fiber with a diameter of 200 μm and a numerical aperture of 0.22 was combined and focused on the gain medium Nd:YAG through an aspherical lens with a focal length of 70 mm. This aspherical lens was mounted on a moving table that could precisely adjust all three axes. The Nd:YAG crystal used as a gain medium was a cylindrical crystal doped with Nd^3+^ 1.0% (Castech Inc., Fujian, China) having a diameter of 5.0 mm and a length of 20 mm. Mirror M_1_, which was the pumping surface of the crystal, was a total-reflection mirror with high reflectivity at 1064 nm (the fundamental wavelength) and high transmission at 808 nm (the pumping wavelength), and the opposite side of the mirror was coated with an anti-reflection coating at 808 nm and 1064 nm. To improve the heat transfer, it was wrapped with 0.2-mm-thick indium foil and mounted on a copper mount. A constant temperature (20 °C) was maintained at all times using thermoelectric cooling modules capable of precise temperature control. 

The AO Q-switch device (AS041-10GSO, Gooch & Housego Inc., Ilminster, UK) using the AO effect was an anti-reflection coated at 1064 nm on both sides, and a quartz crystal with a length of 30 mm was used. It was driven using a 41 MHz and 20 W driver (MQC041-230DC-FPS-15V, Gooch & Housego Inc., Ilminster, UK). Cr:YAG (Castech Inc., Fujian, China), a passive Q-switch device, and a saturable absorber with an initial transmittance of 65% and size of 3 × 3 × 2 mm^3^ were anti-reflection coated on both sides for a wavelength of 1064 nm. Mirror M_2_—with a radius of curvature of 500 mm—and flat mirror M_3_ were high-reflection coated (>99.5%) for obtaining a laser oscillation wavelength of 1064 nm. The angle between the gain medium and mirrors M_2_ and M_3_ was set to the smallest possible value to minimize the astigmatism at 13.3°. Mirror M_4_ was an output flat mirror with a transmittance of 5% for a wavelength of 1064 nm. The distance from the gain medium Nd:YAG to mirror M_2_ was 274 mm, the distance between mirrors M_2_ and M_3_ was 390 mm, and the distance between mirrors M_3_ and M_4_ was 250 mm. These distances are optical distances, considering the refractive index of the optical element inserted into the resonator. The AO Q-switch, which was an AO device, and the saturable absorber Cr:YAG were placed between the gain medium and mirror M_1_. To compare the output pulse width, the saturable absorber Cr:YAG was mounted on the moving table for easy attachment and detachment. The Z-type resonator was configured to reduce the thermal lens effect that occurs in the laser gain medium owing to the high-power pumping laser and to obtain a new laser wavelength of 532 nm for use in PA image realization. This was in order to secure a space for inserting nonlinear crystals, such as lithium triborate (LBO, LiB_3_O_5_) or potassium titanyl phosphate (KTP, KTiOPO_4_). The 532 nm wavelength laser can be realized with nonlinear crystals using intracavity second-harmonic generation.

### 2.2. Output Characteristics of the Doubly Q-Switched 1064 nm Nd:YAG Laser

**Figure 2:**
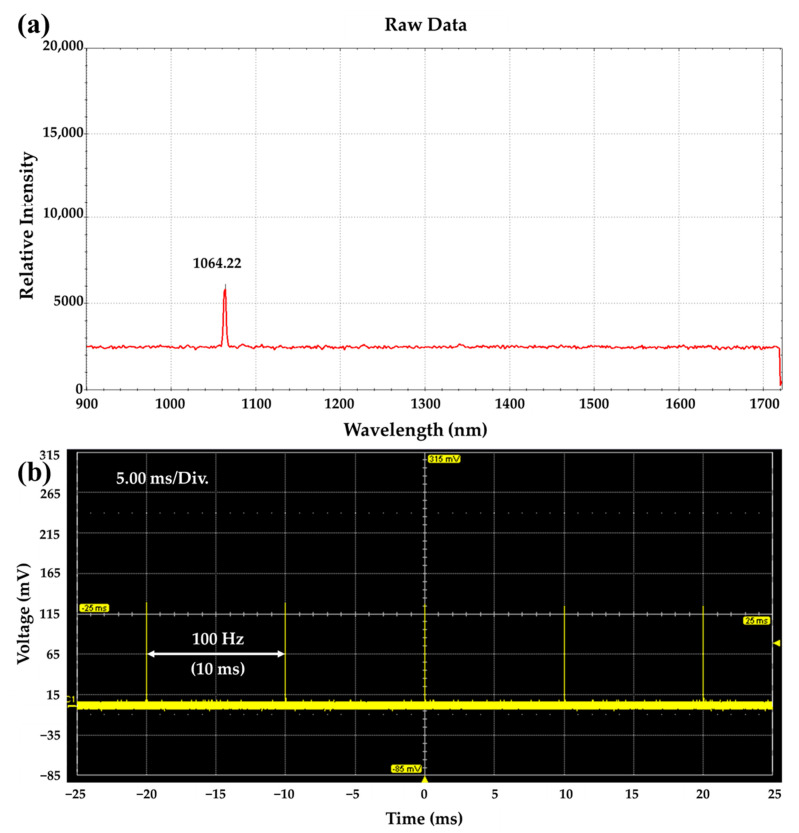
(**a**) Measured spectrum of the doubly Q-switched 1064 nm Nd:YAG laser, (**b**) pulse train of the AO Q-switched 1064 nm Nd:YAG laser.

The pulse repetition rate of the doubly Q-switched 1064 nm Nd:YAG laser developed for PA image applications can be determined within the optimal operating frequency of commonly used AO Q-switch devices. As the repetition rate of the pulse laser used in this study was 100 Hz, the AO Q-switch operating frequency of the developed Nd:YAG laser was fixed at 100 Hz. Figure 2a shows the measured spectrum of the doubly Q-switched 1064 nm Nd:YAG laser using an optical fiber-coupled spectrometer (Sol 1.7, BWTEK Inc., Plainsboro, NJ, USA). The center wavelength and linewidth were measured to be 1064.22 nm and about 5 nm, respectively. Figure 2b shows the pulse train of an oscillating Q-switched Nd:YAG pumped by 808 nm LD fixed at 10 W. The pulse train and pulse width were measured by using an InGaAs PIN detector with a rise time of 175 ps (ET-3000, Electro-Optics Tech. Inc., Traverse City, MI, USA) and an oscilloscope at a sampling frequency of 600 MHz and sampling rate of 2.5 GS/s (WaveSurfer 64Xc, Teledyne LeCroy’s Corp., Chestnut Ridge, NY, USA). As shown in Figure 2b, an AO Q-switched pulse with a stable output at 100 Hz was generated.

**Figure 3:**
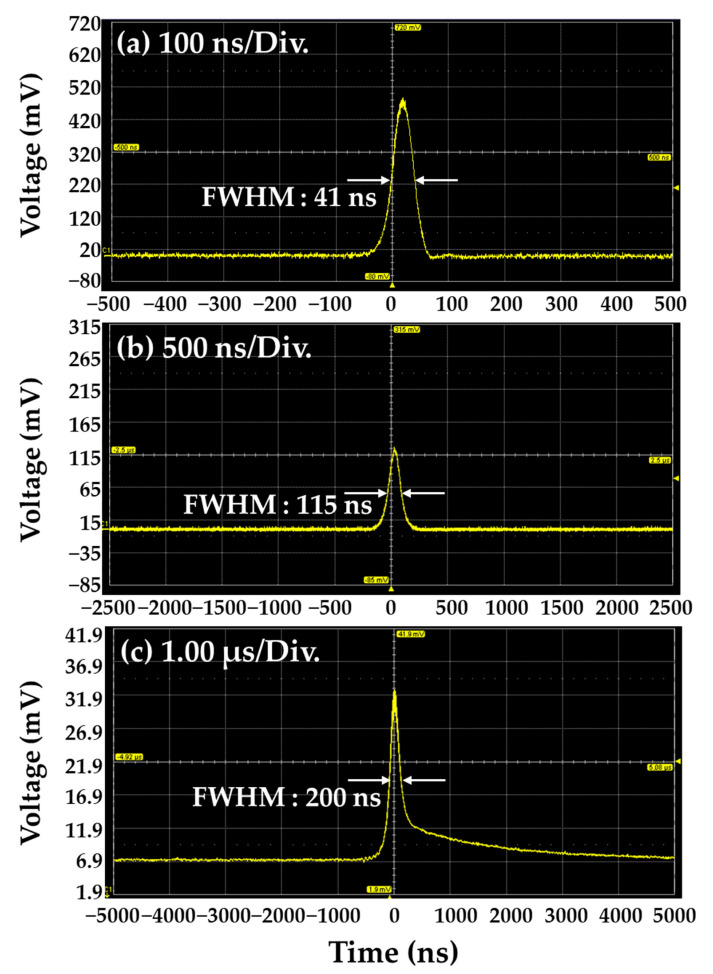
Comparison of single pulse signals for (**a**) doubly, (**b**) AO, (**c**) Cr:YAG passively Q-switched 1064 nm Nd:YAG laser.

The clarity of the PA image is sensitive to the pulse width rather than the laser energy used. Therefore, it is preferable to use pulses as short as possible. If an active AO Q-switch device is used, the laser can be operated with a stable pulse repetition rate, but the oscillating pulse width is long (≥100 ns) owing to the device characteristics. In addition, the passive Q-switch using Cr:YAG saturable absorber has a disadvantage in that the pulse width varies depending on the pump power or the relaxation time of the used saturable absorber and the resonator length, and the frequency cannot be arbitrarily adjusted. Figure 3 shows the single pulse width for different cases. Figure 3a shows a result obtained by measuring the doubly Q-switched pulse in which both Cr:YAG and AO Q-switch were inserted into the resonator, and a pulse width of 41 ns was obtained. Figure 3b shows a result obtained by measuring the AO Q-switched pulse in which the Cr:YAG saturable absorber (a passive Q-switch element in the resonator) was removed and only the AO Q-switch was inserted into the resonator. At this time, a pulse width of 115 ns was obtained. Figure 3c shows a result obtained by measuring the passively Q-switched pulse in which only Cr:YAG was inserted into the resonator, and a pulse width of 200 ns was obtained. Compared to the actively Q-switched 1064 nm Nd:YAG laser by inserting only an AO Q-switch, the doubly Q-switched 1064 nm Nd:YAG laser provides shorter pulse width by 1/3. Therefore, we expect that a clear PA image could be obtained. For experimental conditions, the output power of the 808 nm pump LD was fixed at 10 W, and the operating frequency of the AO Q-switch was fixed at 100 Hz.

**Figure 4:**
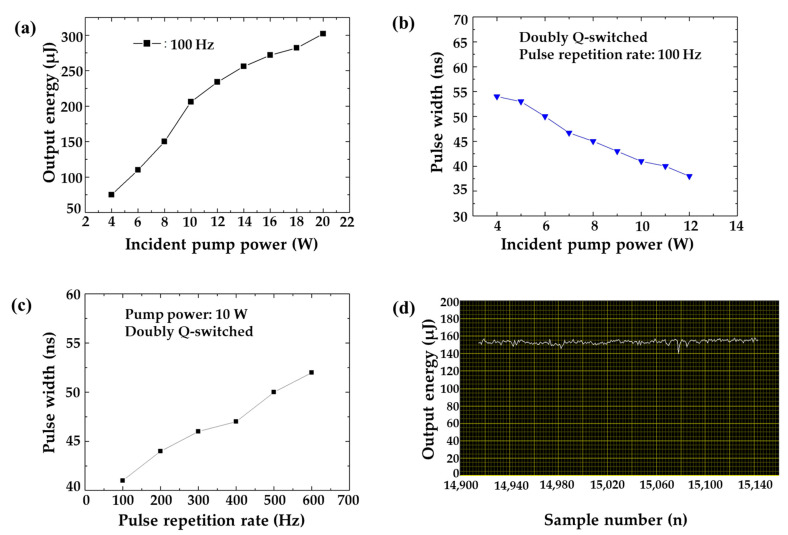
Characteristics of developed doubly Q-switched 1064 nm Nd:YAG laser. (**a**) Comparison of output energy according to incident pump power, (**b**) comparison of output pulse width according to incident pump power, (**c**) comparison of output pulse width according to pulse repetition rate, (**d**) stability of output energy, according to the sample numbers.

Figure 4 shows the characteristics of the developed doubly Q-switched 1064 nm Nd:YAG laser. The output energy was measured with an energy meter (CA/MACH6, Gentec Inc., Quebec City, QC, Canada) and the pulse width was measured with the oscilloscope (WaveSurfer 64Xc, Teledyne LeCroy’s Corp., Chestnut Ridge, NY, USA). Figure 4a is the output energy according to the incident pump power. As the output power of the pump LD was increased, the output energy of the oscillation laser also was increased, and the output energy was measured to be 200 μJ for the LD pumping power of 10 W. Figure 4b is of the output pulse width according to the incident pump power. We can confirm that the output pulse width was decreased as the incident pump power was increased, which is a typical characteristic of the passive Q-switch operation. Figure 4c is of the output pulse width according to the pulse repetition rate. As the repetition rate of the Q-switch operation was increased, the pulse width was increased. From this result, so we can confirm that the pulse width change is not very large, about 10 ns. Figure 4d shows the stability of output energy measured by an energy meter when the repetition rate and LD pump power were 100 Hz and 8 W, respectively. The maximum value of the pulse energy was 1.581 × 10^−4^ J, the minimum value was 1.402 × 10^−4^ J, and the average value was 1.533 × 10^−4^ J, indicating the output energy stability with 8.5%.

### 2.3. System Configuration and Data Process for PA Imaging System Implementation

**Figure 5:**
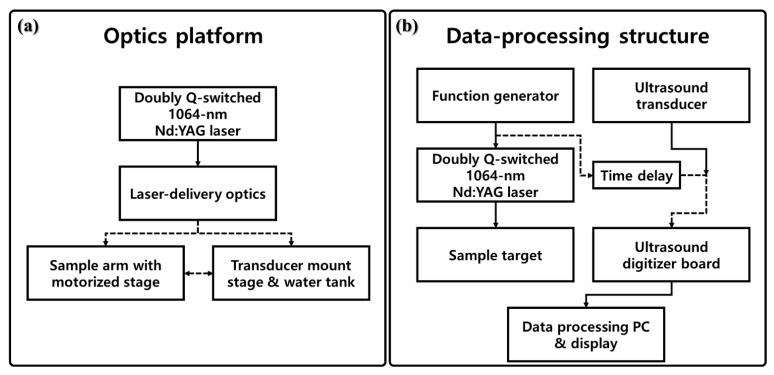
Connection structure for the modules of the implemented PA imaging system: (**a**) optics platform and (**b**) data-processing structure.

We constructed an imaging system to obtain PA signals using the doubly Q-switched 1064 nm Nd:YAG laser. The connection structure for the modules of the implemented PA imaging system is shown in Figure 5.

Figure 5a shows the entire optical system of the implemented PA system, which is divided into four areas: a doubly Q-switching laser unit, an optical delivery unit, a sample moving stage optical system, and a transducer mount stage and water tank. Each area was implemented to enable dynamic movement. In Figure 5a, a dotted black line indicates that the corresponding area was configured to be movable. 

Figure 5b shows the data-processing structure of the implemented PA imaging system. It is driven by synchronous control between the developed laser and the transducer for PA reception using the pulse signal generated by the function generator (DG535, Stanford Research Systems Inc., San Jose, CA, USA) as a system operation-oriented trigger. The black dotted line represents the synchronous control signal that is fluidly driven by a time delay. The obtained PA signal is time-delayed according to the PA signal processing in the function generator (DG535). Subsequently, the signal is amplified using a 36 dB preamplifier (AU-1525, L3 Narda-MITEQ Inc., Hauppauge, NY, USA) and then transferred to a digitizer board (Gage Inc., Lockport, IL, USA).

### 2.4. Developed Doubly Q-Switched 1064 nm Nd:YAG Laser Linked PA System

**Figure 6:**
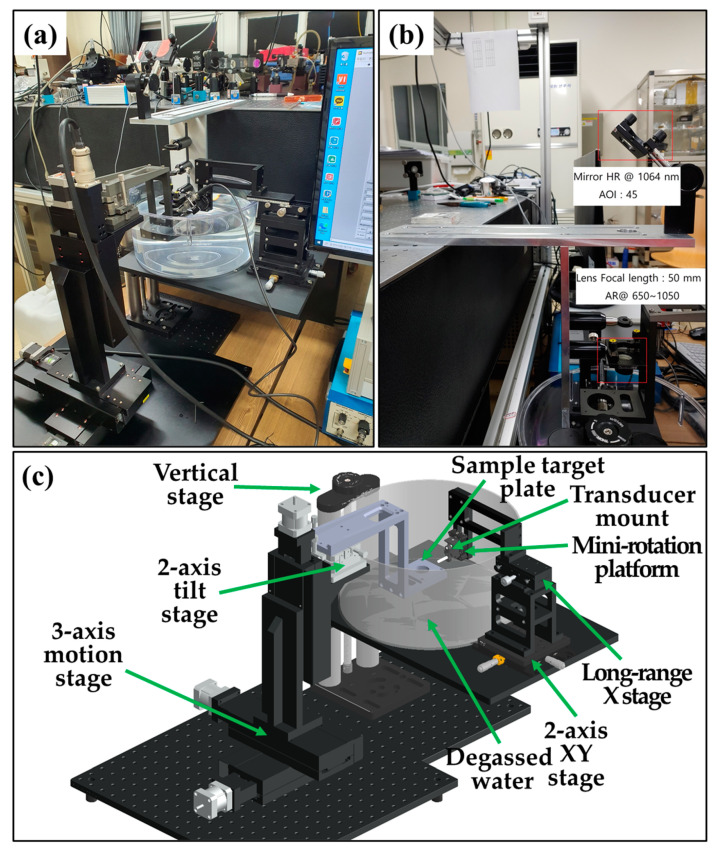
Implemented PA imaging system: (**a**) optical system combined with each developed area; (**b**) laser-delivery optics; (**c**) overall structure of the fabricated sample stage.

Figure 6 presents the configuration of the implemented PA imaging system. Figure 6a shows the entire optical system combined with each developed area, and Figure 6b shows the laser-delivery optics moving from the developed doubly Q-switched 1064 nm Nd:YAG laser to the sample stage. Laser-delivery optics changes the optical path of the developed laser to a sample stage and consists of optical components for focusing the light energy on the sample target. A lens with a focal length of 50 mm (ACA254-050-B, Thorlabs Inc., New Jersey, NY, USA) was used as the objective lens of the laser incident on the sample.

Figure 6c shows the overall structure of the fabricated sample. As shown on the left side of Figure 6c, the sample arm with a motorized area was implemented separately with a 500 × 500 mm^2^ aluminum breadboard. As shown on the right side of Figure 6c, the transducer mount stage and water tank were composed of a 300 × 300 mm^2^ aluminum breadboard. In the transducer mount stage and water tank area, the vertical stage (VAP10/M, Thorlabs Inc., New Jersey, NY, USA) was used to adjust the water tank height (~254 mm), the transducer mount (VC1/M, Thorlabs Inc., New Jersey, NY, USA) was used to change the thicknesses (0.5–20.8 mm) of the transducer, the mini-rotation platform (MSRP01/M, Thorlabs Inc., New Jersey, NY, USA) was used to adjust the transducer angle in the sample direction, the long-range (±30 mm) X stage (XDTS90, Impsystem Inc., Gumi, Korea) was used to move the transducer to sample plate, and a 2-axis XY stage (XYT1/M, Thorlabs Inc., Newton, NJ, USA) was responsible for the fine adjustment of the transducer position (~13 mm). The sample image experiment was performed by positioning the sample target and adjusting the vertical stage in a water tank filled with degassed water.

The sample arm with a motorized stage area consisted of a plate on which the sample target, a 3-axis motion stage, and a 2-axis tilt stage (LP-NOSO2, Impsystem Inc., Gumi, Korea) were mounted. The 2-axis tilt stage (±3°) was used to improve the signal-to-noise ratio when acquiring an acoustic signal from a transducer. A 3-axis motion stage (SM3, Sciencetown, Seoul, Korea) was used for image acquisition of the sample target. The motion control unit of the 3-axis motion stage was controlled using a motion control box (PMC-1HS and PMC-2HS, Autonics, Incheon, Korea). 

The fabricated sample stage has an allowable thickness of 0.5 to 20.8 mm for a single ultrasound transducer and an adjustable angle of 360° degrees. In addition, the position of the transducer can be adjusted by up to 73 mm in the x-axis and 13 mm in the y-axis by two translation stages (long-range X stage, 2-axis XY stage).

In the scan performance by motion control, the maximum scan range is 150 mm for each of the x-, y-, and z-axes, the minimum control distance is 2 μm, and the maximum scan speed is 10 mm/s.

## 3. Results and Discussion

### 3.1. Doubly Q-Switched 1064 nm Nd:YAG Laser

In laser sources for PA systems, the adjustable pulse repetition rate, pulse width, pulse energy, and pulse power are the main parameters. Therefore, Table 1 presents the specifications of the doubly Q-switched Nd:YAG laser developed for PA imaging system applications.

In general, as the wavelength of the laser is diversified in the PA system application, an extended research field can be conducted. The developed doubly Q-switched Nd:YAG laser has the advantage that it can be utilized through various wavelength conversions in addition to the main oscillation wavelength through some modifications.

The developed doubly Q-switched laser, which is suitable for PA systems, can be used as a light source with a wide wavelength range. The main oscillation wavelengths of the developed Nd:YAG laser are 1064 nm (^4^F_3/2_ → ^4^I_11/2_ transition), 946 nm (^4^F_3/2_ → ^4^I_9/2_ transition), and 1319 nm (^4^F_3/2_ → ^4^I_13/2_ transition), and they can be easily converted to RGB light sources such as 532, 473, and 660 nm, which are the second harmonics of these wavelengths. Therefore, the laser has the advantage of applying light sources of various wavelengths to PA image acquisition. In the future, we plan to broaden the coating area of mirrors M_1_, M_2_, and M_3_, as well as AO Q-switched elements, including the mirror of interchangeable type M_4_. Therefore, the proposed customized doubly Q-switched laser-based PA imaging system can be modified for more diverse conditions, including the wavelength, frequency, pulse width, and sample target.

### 3.2. Image Acquisition Results for Developed PA Imaging System

**Figure 7:**
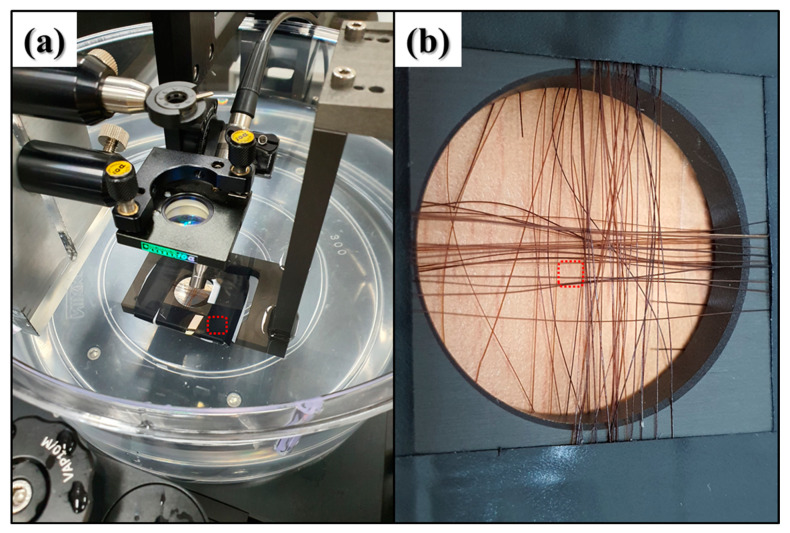
Experimental setup for PA imaging: (**a**) experimental setup; (**b**) sample target used.

The setup for the image acquisition experiment using the PA system is shown in Figure 7a. The sample target for image acquisition was constructed by drilling a 1-inch hole into a solid aluminum plate. The hair was fixed around the hole on both the x- and y-axes. It was attached to the plate using an insulating tape to determine the focal position and examine the PA signal.

Figure 7b shows the fabricated target sample. The experiment was performed using a manufactured sample target, as shown in Figure 7a, which was mounted on a sample arm with a motorized stage. The sample was scanned while moving the motorized stage along the input step distance.

**Figure 8:**
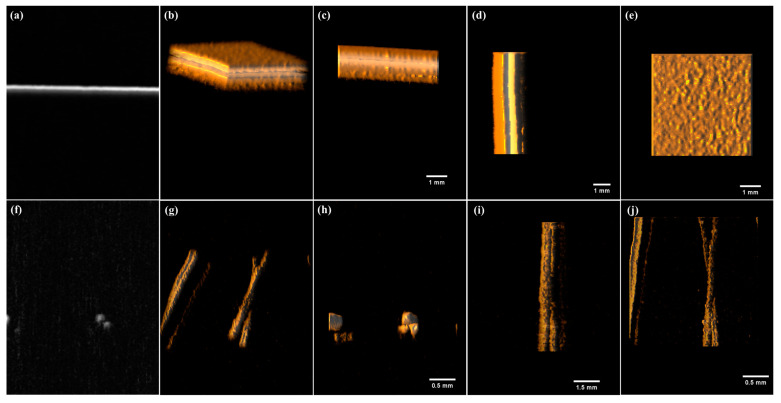
Experimental results for the PA imaging system obtained using insulating tape and hair: (**a**) original imaging result for insulating tape; (**b**) 3D reconstruction result for insulating tape; (**c**) insulating tape imaging result view of x- and y-axes; (**d**) result view of y- and z-axes; (**e**) result view of x- and z-axes; (**f**) original imaging result for the hair; (**g**) 3D reconstruction result for the hair; (**h**) hair imaging result view of x- and y-axes; (**i**) result view of y- and z-axes; (**j**) result view of x- and z-axes.

As shown in Figure 7, the image acquisition experiment using the PA system was conducted in two areas: the first area where the hairs are separated because of their height differences and intersections, and the second area where insulating tape is superimposed. In Figure 7a,b, the area photographed in the experiment is indicated by the red dotted lines. A 15 MHz and 0.25 inch focused ultrasound transducer (Olympus Inc., Shinjuku, Toyko, Japan) was used. Figure 8 shows the processed image results for the obtained PA signals. 

Figure 8a–e shows the image results for the insulating tape sample superimposed onto the attached insulating tape. For experimental conditions, the motorized stage was moved to 100 numbers of 50 μm step size within a section of 5 mm for each x- and y-axis PA signal. Figure 8f–j shows the processed image results for the hair samples. For experimental conditions, the motorized stage was moved to 250 numbers of 10 μm step size within a section of 2.5 mm for each x- and y-xis PA signal. Figure 8a,f shows the obtained original images processed from the PA signals. Figure 8b–e,g–j shows images regenerated in the ImageJ 1.53k program after applying normalization and a median filter with the acquired PA signal. Figure 8b,g shows the reconstructed three-dimensional (3D) image results. In Figure 8b, the adhesive surface can be distinguished for the overlapping insulating tape sample target. In Figure 8g, the two hair samples can be distinguished in the height difference and intersection area for the hair-sample target. Figure 8c–e,h–j shows the scales of the imaging area for the insulating tape and hair samples. The results of each sample for the x- and y-axes, y- and z-axes, and x- and z-axes are shown.

**Figure 9:**
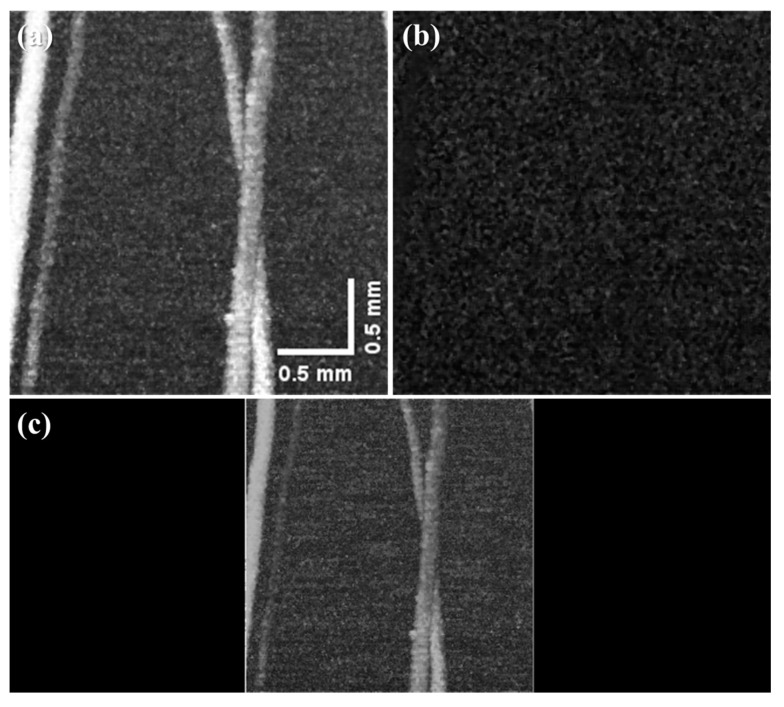
Reconstructed hair PA image results: (**a**) MIP image result; (**b**) PA image result by depth; (**c**) 3D rotation view of the hair PA image result; (**b**,**c**) are video clips (ref. attachment file).

Figure 9a–c shows the maximum intensity projection (MIP) image of the hair-sample target, the image on the depth change, and the sample image for each position with a 3D motion view, respectively.

## 4. Conclusions

We developed a customized doubly Q-switched laser that can control the pulse width to easily find weak acoustic signals in PA systems. As the laser was developed using an AO Q-switch, in contrast to the existing commercial laser system, it is relatively easy to control the pulse repetition rate and pulse width. Therefore, the developed doubly Q-switched laser has a wide range of pulse repetition rates (10 Hz–10 kHz) and an adjustable pulse width (40–150 ns) suitable for a PA imaging system. A custom-made multiple axes stage was used to develop a fully customized PA system.

A custom-made sample stage with a nine-axes control unit design (five axes for the sample arm module and four axes for the transducer-implement module) for the PA system allows the sample target and transducer to be individually and freely adjusted (sample target: x-, y-, and z-axis distance each up to 150 mm and tilt angle up to ±3°, transducer: thickness 0.5~20.8 mm, adjustable angle 360°, adjustable position: up to 73 mm for x-axis and 13 mm for y-axis). Additionally, the optical system in the implemented PA imaging system was designed in a structure that can be freely modified and converted according to the sample target with modularization. Therefore, making it suitable for capturing weak PA signals. Finally, the performance of the developed PA system is 150 mm in each of the x-, y-, and z-axis, the minimum travel distance is 2 μm, and the maximum scan speed is 10 mm/s for the sample target.

The developed doubly Q-switched laser suitable for PA systems has a similar price to commercial lasers at 1064 nm single wavelength, but with the advantage of being customized, the oscillation wavelength can be changed relatively easily using the characteristics of the Nd:YAG laser. Therefore, the developed doubly Q-switched laser has a cost advantage in laboratory-level research over using other multi-wavelength light sources as light sources for PA systems. Moreover, we expect that our proposed technique will be helpful in conducting basic and applied research for PA imaging system applications.

## Figures and Tables

**Table 1:** Specifications of the developed doubly Q-switched 1064 nm Nd:YAG laser.

Parameter	Specification
Wavelength	1064 nm
Pulse repetition rate	10 Hz–10 kHz
Pulse width (full width at half maximum)	40–150 ns
Pulse energy	50–500 μJ
Pulse power	100–1000 mW at 1 kHz

## Data Availability

The data presented in this study are included within the article.
